# Residual Inequity: Assessing the Unintended Consequences of New York City’s Clean Heat Transition

**DOI:** 10.3390/ijerph15010117

**Published:** 2018-01-11

**Authors:** Daniel Carrión, W. Victoria Lee, Diana Hernández

**Affiliations:** 1 Department of Environmental Health Sciences, Mailman School of Public Health, Columbia University, 722 W. 168th Street—11th Floor, New York, NY 10032, USA; w.victoria.lee@cantab.net; 2 Department of Sociomedical Sciences, Mailman School of Public Health, Columbia University, 722 W. 168th Street—9th Floor, New York, NY 10032, USA; dh2494@cumc.columbia.edu

**Keywords:** clean heat, home heating oil, urban health, energy policy, health equity

## Abstract

Energy policies and public health are intimately intertwined. In New York City, a series of policies, known as the Clean Heat Program (CHP), were designed to reduce air pollution by banning residual diesel fuel oils, #6 in 2015 and #4 by 2030. This measure is expected to yield environmental and public health benefits over time. While there is near-universal compliance with the #6 ban, a substantial number of buildings still use #4. In this paper, geographic analysis and qualitative interviews with stakeholders were used to interrogate the CHP’s policy implementation in Northern Manhattan and the Bronx. A total of 1724 (53%) of all residential residual fuel burning buildings are located in this region. Stakeholders reflected mostly on the need for the program, and overall reactions to its execution. Major findings include that government partnerships with non-governmental organizations were effectively employed. However, weaknesses with the policy were also identified, including missed opportunities for more rapid transitions away from residual fuels, unsuccessful outreach efforts, cost-prohibitive conversion opportunities, and (the perception of) a volatile energy market for clean fuels. Ultimately, this analysis serves as a case study of a unique and innovative urban policy initiative to improve air quality and, consequently, public health.

## 1. Introduction

Energy policy represents one of the many opportunities to simultaneously influence environmental sustainability and public health in an increasingly urbanized world. New York City (NYC) has been a laboratory for such initiatives, particularly under the Bloomberg Administration (2002–2013). A seminal outcome of the administration’s proactive efforts to protect public health and the environment was the inaugural *PlaNYC report* published in 2007. This report highlighted three major challenges facing NYC: population growth, an aging infrastructure, and a precarious environment. The environment section of the report pointed out that, despite improvements, NYC continuously failed to attain the minimum criteria for federal Clean Air Act standards, specifically for ozone and PM_2.5_ [1]. Indeed, NYC’s poor air quality had also been highlighted in the *State of the Air* report issued in the same year by the American Lung Association, which showed similarly negative results for NYC (Table 1). Yet since 2007, NYC has made great strides to improve the air quality. Table 1 also shows how NYC’s Air Quality has improved substantially over the past decade. While ground-level ozone remains a concern, particulate concentrations have decreased notably.

This paper highlights and analyzes one Bloomberg era initiative which undoubtedly contributed to improved air quality, the Clean Heat Program. The NYC Clean Heat Program was an innovative policy initiative which aimed to reduce air pollution and its health consequences by decreasing pollutant emissions from buildings’ boilers. The policy mandated transitions from residual diesel fuel oils to cleaner-burning alternatives. Such a program is worth studying because it entailed a complex collaboration of government, private, and non-profit actors toward a common goal of air quality improvement. However, the initiative was not without its faults. Our study precipitated from the realization that a substantial proportion of buildings were still burning residual fuel oil in Northern Manhattan (north of 110th Street) and the Bronx. At the time of our study, these areas bore a significant burden of buildings that had not yet transitioned to cleaner burning fuels, with 1724 (53%) of the yet-to-convert buildings located in this region [4]. This is of particular concern given the historically poor health and social disadvantage of residents in Northern Manhattan and the South Bronx, herein referred to as Uptown. For example, Uptown residents demonstrate some of the highest asthma rates in the entire country [5]. The area is also predominantly populated by people of color, and of lower socioeconomic status, as indicated by higher poverty concentrations [6]. Our analysis is largely focused on evaluating outcomes associated with this policy from an environmental justice perspective. We do so by mapping the spatial and demographic trends associated with policy compliance, and further examine the policy implementation process via stakeholder interviews and relevant policy documents. The overall objective of this study is to understand the barriers and facilitators to the Clean Heat program implementation in environmental justice communities. 

## 2. Background and Literature Review

The 2007 *PlaNYC* report established an ambitious goal: to “achieve the cleanest air quality of any big city in America” [1]. Fourteen strategies were outlined in order to improve air quality. “Energy efficiency/heating” represented the largest category of opportunity for PM_2.5_ emissions reduction. Specifically the tenth strategy sought to “promote the use of cleaner burning heating fuels” by targeting residual fuel oils #4 and #6 [1]. 

Residual fuel oils are diesel-based fuels that remain after gasoline and distillate fuels have been removed from crude oil [7]. Research shows that residual fuels produce higher levels of particulate matter (PM) than any other diesel type, largely due to their elevated sulfur composition [8]. The NYC Department of Health and Mental Hygiene (NYC DOHMH) estimated that even a 10% reduction in PM_2.5_ would save hundreds of lives annually, yet NYC remained one of the only municipalities in the country that still allowed the use of residual fuel oil #6 [9]. Of the approximated 10,000 buildings that burned residual fuel oils in 2007, roughly 6000 burned #6 while 4000 burned #4 and were dispersed throughout Manhattan (mostly) and other parts of the city [1]. Residual fuel oils #4 and #6 had historically been cheaper than ‘cleaner’ options such as natural gas and distillate fuels—likely explaining their continued use in NYC [10,11]. Unfortunately, residual fuels also yield higher pollutant concentrations upon combustion [12]. Air pollutants such as PM, ozone (O_3_), carbon monoxide (CO), sulfur dioxide (SO_2_), lead, and nitrogen dioxide (NO_2_) are all regulated as criteria air pollutants by the Environmental Protection Agency (EPA) via the National Ambient Air Quality Standards and their health impacts are widely acknowledged [13]. Two lesser known, but nonetheless harmful, air contaminants are nickel and vanadium. These substances have exhibited strong positive associations with cardiovascular disease in laboratory and population studies [14]. Nickel and vanadium are of lesser concern nationwide because they remain at significantly smaller concentrations outside of the NYC Metropolitan area [14]. Scientists concluded that the continued use of residual fuel oils were the major contributor to elevated nickel and vanadium levels in NYC [15]. Beyond the obvious health benefits from reducing PM_2.5_, nitrous oxides (NO_x_), nickel, and vanadium, the reductions would likely have the added an opportunity for greenhouse gas mitigation [16,17]. 

Three policies were used to improve ambient air quality in NY (Table 2). One local regulation change, one local law, and one state law together served to form the NYC Clean Heat Program [4,18]. While any one policy can easily stand alone, the Bloomberg Administration was unlikely to reach the targets set forth in *PlaNYC* without a coordinated policy effort. Together, elected and appointed officials, advocacy groups, and citizens collaborated to enact a suite of clean heat policies.

*Clean Heat Program Implementation:* The NYC Mayor’s Office of Long-term Planning and Sustainability was the main government office coordinating the Clean Heat Program, but worked closely with the Environmental Defense Fund. There were also extensive partnerships with utilities companies/providers such as the Consolidated Edison Company of New York, National Grid, and New York Oil Heating Association [4]. The goal of these partnerships was to increase the availability of natural gas and #2 diesel throughout NYC [19]. A task force was also formed to represent community partners, industry, local government agencies, and unions. A Clean Heat Program website provided access to technical assistance, incentives for fuel-efficiency upgrades and cleaner burning fuels, and a resource list of professionals licensed to perform boiler upgrades in NYC. In addition, the program made available case studies, testimonials, and “how to” guides [4]. A clear emphasis was made on outreach and education. 

Financing incentives were also built into the program because it was widely acknowledged that boiler upgrades could be costly for some buildings, and these financing programs were implemented to bridge that divide by offering additional resources to property owners [11,19]. Clean Heat collaborated with banks, including traditional and green lenders, to make loans readily available while the city also created a $5 million loan loss reserve to increase availability to low-income buildings [19]. Financing tools include access to mortgages, equipment lenders whereby specific equipment is financed, and establishing energy service agreements that outlined extended commitments to particular energy companies in return for paid upfront conversion costs [4]. 

Our research aimed to understand the effectiveness and any unintended consequences of this policy shift on environmental justice communities. Existing research continues to demonstrate disproportionate impacts of environmental contaminants on disadvantaged communities, but little work has focused on the factors mediating disadvantage and differential exposures. Unequal policy implementation represents one possible mediating pathway, and was the focus of our mixed methods case study of clean heat policy implementation in NYC.

## 3. Methods

The focus of this analysis is on residential building transitions to cleaner burning fuel sources, primarily because this is the largest proportion of the building stock in NYC. Non-residential buildings were omitted from this analysis because of possible differences in resource availability, incentive structures, and policy responsiveness. Our study of the Clean Heat Program implementation in Uptown residential buildings is based on primary and secondary data sources: (1) quantitative data exploration and mapping of secondary source data; and (2) qualitative analysis of stakeholder interviews interwoven with document analysis of relevant literature to ground stakeholder perspectives. This study followed informed consent procedures approved by the Columbia University Institutional Review Board. 

*Spatial and quantitative data:* Secondary source data were explored for spatial and demographic trends of heating oil transitions in order to assess potential inequities and explanations for these relationships. We downloaded all quantitative data from NYC government websites. Since our interest was in residential heating fuel transitions, we used two datasets to understand the impacts of the Clean Heat Program. The first dataset was from the NYC Open Data website, labeled ‘Oil Boilers—Detailed Fuel Consumption and Building Data’, created in September 2011 [20]. The second dataset was from NYC Clean Heat’s *Spot the Soot* website (https://www.nyccleanheat.org/spot-the-soot) [4], downloaded soon after its final update in November 2015. In fact, the Spot the Soot website provided more than just data, but also had a Google Map with the location and fuel type of residual fuel burning buildings, acting almost as a surveillance tool. These datasets represent snapshots in time, only indicating if a building was burning a residual fuel and whether it was #4 or #6 diesel. Merging the two datasets allowed us to assess patterns in heating oil transitions. Geoprocessing was conducted by attribute joining building information from the boiler datasets to NYC tax lot maps known as MapPLUTO [21]. Demographic data for NYC census tracts were downloaded from the 2015 American Community Survey five-year estimates and shapefiles at the same administrative level were retrieved from the NYC Open Data portal. Spatial joins were used to connect Clean Heat building-level data to census tracts. All geoprocessing and mapping was conducted in QGIS 2.18 (QGIS Development Team, www.qgis.org) while data manipulation, summary statistics, and figures were produced with R 3.2 (R Core Team, Vienna, Austria). 

*Qualitative interviews:* The primary purpose of the qualitative phase of the study was to understand how the clean heat policy emerged and unfolded since being enacted in 2011. We gathered the perspectives of policy makers and those responsible for implementing the law as well as the role of other stakeholders including service professionals and building owners/managers subject to the mandated changes. The rationale for these interviews was rooted in the need to: (a) understand how policies such as this develop, (b) document process-level factors in its implementation; and (c) identify best practices and lessons learned. We conducted 10 in-depth, semi-structured interviews with key informants who were responsible for motivating and carrying out the policy. We examined their knowledge and perceptions of the Clean Heat Program, as well as their unique perspective in the implementation of the policy. The 10 key informants included “policy makers” consisting of a highly-ranked public official overseeing a city agency responsible for regulating the policy, a resident-advocate, a representative from the office of a local elected official, and two staffers at community-based non-profit organizations contracted to conduct outreach efforts and program support. Policymakers provided information on the historical context for the development of the Clean Heat regulations and the various factors and experiences involved in the passage of the law. They also shared their knowledge, perceptions and practical considerations on the use of the clean heat law for environmental and public health purposes. We also interviewed “policy implementers”, including two building owners/managers of multiple residential apartment buildings subject to the fuel transition, an independent heating oil distributor, and two bank lenders specializing in green development. Policy implementers provided practical information about needs and challenges of building owners/managers as it relates to compliance and also considerations, perceptions, and practices in the conversion process. Building owners/managers and residents provided information on the lived experience of the law. This small, yet purposive, sample of assorted key informants provided a comprehensive overview of the Clean Heat Program planning and implementation process. The sample was strategically selected due to the highly specialized nature of their knowledge and experience and the respondents’ ability to provide answers to process-level questions related to the policy. Participants were recruited directly through the Clean Heat Program advisory council and also by referral from partner organizations in the study area, or elected/appointed officials familiar with implementation of the program in Uptown. All interviews were semi-structured and targeted to the area of expertise of each stakeholder. Topics covered in interviews with key informants included (a) perception of clean heat and boiler conversion; (b) attitudes towards clean heat regulations; (c) understanding of the building conditions including and beyond the boiler; (d) other conservation activities or maintenance upgrades completed in this process; (e) economic issues including the cost of conversion, the finance process and plans for the absorption of costs; (f) perceptions of residents in this process; (g) perceptions of support, efficiencies and efficacy in the process; and (h) recommendations for improving the process (i.e., communication, financing, administrative delays, etc.). 

The questions were flexible, and the progress of the interview was led by topic guides covering pertinent domains of inquiry, developed as part of preliminary research activities for a larger project that examined resident and building-level outcomes associated with the transition to cleaner burning fuels. Interviews were conducted by phone except one was conducted in person. Each session was digitally recorded and lasted about 30–45 min. Recorded interviews were transcribed and checked against the recording for fidelity. All interviewees were anonymized in transcription. 

During the qualitative data analysis phase, we identified patterns of knowledge, perceptions, and practices from stakeholder viewpoints. Using an inductive approach, we explored relevant issues and concerns related to the boiler conversion process. Following the standard conventions of qualitative data analysis, each verbatim transcript was coded in a two-step process in order to examine the overall content and common themes which helped us to identify emergent patterns, as well as to extract the lessons learned and relevant recommendations [22]. Transcripts were analyzed using Atlas.ti version 6.1 (Scientific Software Development GmbH, Berlin, Germany), wherein they were first coded with open codes in order to identify broad themes, followed by more interpretive codes that were used to identify core concepts and patterns in the data. Qualitative analysis of interviews helped to: (1) understand commonly shared concerns, criticism, or praise for the policies; (2) uncover any contradictions between policy intent/justification and stakeholder perspectives; (3) explain gaps in the policy’s logic or approach from document analysis; and (4) assess the effectiveness of the policy to date. 

*Document data:* Spatial/quantitative and interview-based data were complemented by the review and analysis of document-based sources, including city and state legislative and regulatory policy documents, utilities data, and peer-reviewed scientific literature for triangulation purposes. Policy documents were identified from city and state legislative databases, utilities data were readily available on utility company websites, and peer-reviewed scientific literature was found via Google Scholar. Textual analysis and qualitative data from interviews were interwoven in this study to offer a panoramic perspective of the NYC Clean Heat program, as well as to identify the strengths, weaknesses, and opportunities of the Clean Heat Program as a city government initiative. We first present the results of the spatial and quantitative analysis to document geographic and demographic trends in boiler transitions. Results from qualitative analysis of stakeholder interviews and document analysis are then presented together to provide potential explanations for implementation differences in Uptown.

## 4. Results

### 4.1. Unequal Implementation—Geographic and Demographic Trends

**Figure 1:**
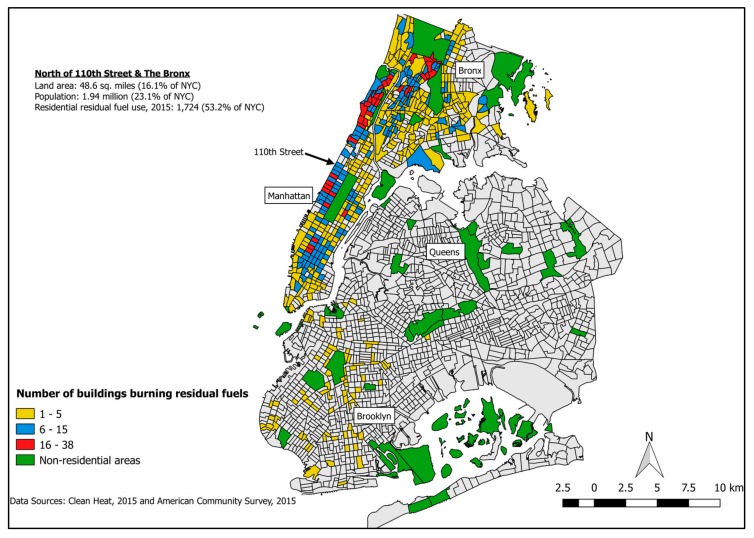
Remaining Residual Fuel Boilers per US Census Tract, 2015.

By November 2015, there was near universal compliance of the #6 ban, with only 19 residential buildings still using this fuel type. Of those 19 buildings, ten were in the Bronx, eight were in Lower Manhattan, and one was in Queens. Exploring the data further uncovered geographic patterns within those residential buildings still using residual fuel oil #4. Most residential buildings that had not yet transitioned to clean heating oils were situated in Uptown, with few remaining in other NYC boroughs (see Figure 1). More specifically, 1724 (53%) of residual fuel-burning buildings were in Uptown.

**Figure 2:**
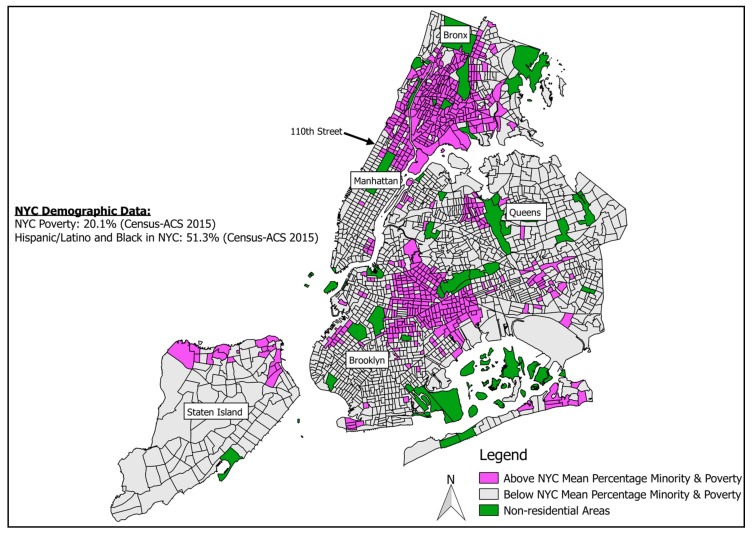
Disadvantaged Populations by U.S. Census Tract (Note: The study defines ‘Uptown’ as above 110th Street and the Bronx).

There is clearly great heterogeneity throughout the neighborhoods located within Uptown but, overall, these neighborhoods are marked by disadvantage. Collectively, of the 1.94 million individuals in Uptown, 30% live in poverty, compared to 20% for all of New York’s 8.25 million inhabitants [6]. Furthermore, Uptown is predominantly comprised of communities of color, with 82% identifying as Black and/or Hispanic/Latino compared to 51% of NYC overall [4]. Uptown also has disproportionately less home ownership, with only 17.4% of housing being owner-occupied compared to 34.5% throughout NYC [6]. Figure 1 shows neighborhoods at the intersection of high poverty (above 20%) and high racial/ethnic minority composition (above 51%). A disparity, therefore, seems apparent when comparing these demographic data to the Spot the Soot data: Figure 2. Uptown Manhattan and the Bronx only represent 16% of the land area of NYC and 23% of the population, but 53% of buildings continuing use of residual fuels as of November 2015.

**Figure 3:**
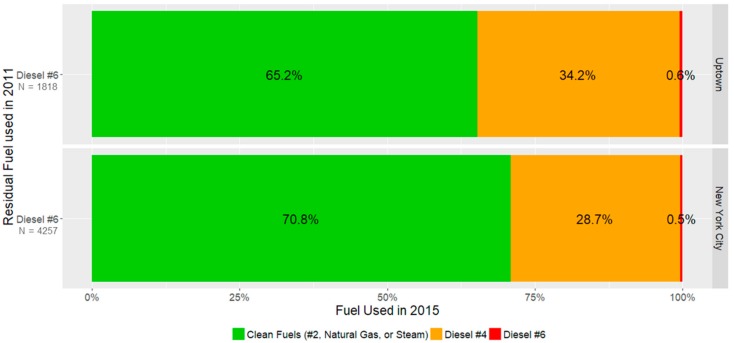
Of buildings burning diesel #6 in 2011, what fuel were they using by 2015?

Proportionally fewer #6-burning buildings in Uptown transitioned to clean fuels between 2011 and 2015 compared to all of NYC. Figure 3 demonstrates that of the 1818 #6 burning buildings in Uptown, only 1186 (65.2%) transitioned to cleaner fuels, whereas throughout NYC, of the 4257 buildings using #6, 3016 (70.8%) transitioned to clean fuels.

This means that Uptown buildings were more likely to simply transition to #4 diesel rather than upgrading to a cleaner fuel. Table 3 demonstrates that these differences in transition patterns were sufficient to shift the burden of residual fuel burning buildings from the rest of New York City in 2011 to Uptown in 2015. We explored some additional building-level data between these two groups to assess potential differences between them. Among residential residual fuel burning buildings in 2011, these data reveal no differences in age of buildings or boilers between Uptown and the rest of NYC. However, looking at building size as measured by the total number of units, buildings that converted to cleaner fuels tended to be larger (mean = 89.2) than buildings that continued to use residual fuels (mean = 59.5). This may be a proxy for differences in financial resources available to landlords. 

Acknowledgement of the socio-demographic and spatial trends in heating oil transitions bolstered our interest in identifying patterns in stakeholder perspectives, complemented by document data, which may explain some of the differences in heating oil transitions.

### 4.2. Key Informant Interviews and Document Data

Key informant interviews and document analysis offered insights into three main categories of findings: (1) awareness of air pollution among NYC residents; (2) the process of converting fuel types, including weighing options, decision-making and taking action; and (3) problems associated with compliance, including outreach, awareness, and implementation challenges. 

#### 4.2.1. Awareness

Air pollutants from building boilers, and their consequences, have not gone unnoticed by NYC residents and property owners/managers. A resident-advocate in our study recounted her experience living adjacent to a building using #6 oil:

“…I’m on the top floor. I’d never seen emissions before, and March 2006 were the first emissions that I saw. And there was huge, it was like a volcano of black smoke that came directly into my kitchen window… I realized immediately when it smelled like a city bus, like I was standing behind a city bus that we needed to get the heck out of the apartment… I shut the window but it was too late… I developed asthma in the same year, 2006. And I was 43 years old. I’d never had asthma in my life.”

(Resident-advocate)

Indeed, although evidence is mixed, existing studies have suggested that adult onset asthma may be caused or exacerbated by exposure to ambient air pollutants [23,24]. The same participant went on to explain:

“…People should know about this [residential boiler fuel type], I doubt they know when they buy, they don’t know when they rent. There’s no way for people to know what they’re stepping into. And once you’re there, like I said, almost like blackmail. If you make noise you might lose your apartment…”

(Resident-advocate)

Over the course of the clean heat transition period, NYC’s government hotline (311) received several air quality complaints suggesting public awareness of pollutants, particularly those likely connected to building-level emissions. For instance, in 2009 there were 8601 air quality-related 311 complaints in NYC, of which 1730 were labeled “Air, Smoke, Residential (AA1)” [25]. Furthermore, approximately 2200 complaints were made via 311 concerning boiler smoke from neighboring buildings [26]. In 2015, there were slightly fewer air-quality complaints, 8485 in total, of which 1668 were categorized as “Air, Smoke, Chimney, or Vent” (AS1) [27]. When plotting these complaints geographically, the majority were issued by lower Manhattan residents (825), followed by northern Manhattan and the Bronx (371) and the remainder scattered throughout Brooklyn and Queens (472). These data suggest that the public is aware of boiler emissions and potential concerned about negative consequences. However, despite the disproportionate concentration of buildings burning dirty fuels in Uptown, the complaints were initiated from areas of the city that are arguably more privileged. Disadvantaged New Yorkers may not fully understand the relationship between boiler fuel types and air quality, while those who do may not feel empowered to take action to address these conditions, so as not to jeopardize their housing. 

#### 4.2.2. Process

*Weighing Conversion Options:* The conversion options from residual fuels including transitions to #2 diesel, natural gas, or cogeneration. Natural gas service was largely available throughout the city, with sporadic geographic regions without service during the implementation period—although plans include full coverage by 2019 [28]. However, the availability of cogeneration or natural gas was geographically restricted due to infrastructure issues. Cogeneration is limited to those below 96th street in Manhattan [28]. The vast majority of buildings in NYC, therefore, could only choose between #2 and natural gas as extending pipeline infrastructure is a capital intensive endeavor.

Although ConEdison (the local utility provider) initiated a natural gas zone development program to extend service to new regions, the program required strict compliance and was available only during limited time frame. Even then, many interviewees pointed out that ConEdison often did not adhere to their own timelines. One property manager commented:

“I had one [natural gas hookup] that was more than twelve months late.”

(Building owner)

Several informants opted for dual burners, whereby heating equipment can operate on #2 or natural gas depending on prices and availability. A mortgage lender noted:

“I would never recommend any building sticking with one fuel, because no one ever has been right. The cost of fuel, the cost of gas is going to [fluctuate] over the next 20 years, so I always advocate for full convertibility.”

(Green lender)

Some buildings with dual burners signed up for an interruptible program. This meant that natural gas is their main source of fuel, but they are expected to switch to another fuel source upon the supplier’s request or when temperatures dip below a certain temperature. Challenges arise when the interrupted building may suddenly need service from an oil purveyor. These calls may be unanticipated and emergent. A representative for heating oil companies further explained,

“So if those interruptibles come on, and those 4000 accounts cost me 2 million gallons, which I did not know I was going to have to use…the [oil] price spikes.”

(Heating oil supplier)

Although many individuals believed that natural gas was better for the environment, a home heating oil supplier contended that:

“…ultra-low Sulfur #2 has close to zero soot emissions, the lowest of all conventional heating fuels.”

(Heating oil supplier)

This perspective was shared by a green mortgage lender, who noted issues on the supply side of the clean energy equation:

“Not everybody is a fan of natural gas, ultra-low Sulfur diesel #2 is certainly even a cleaner source of energy than natural gas, without the implications that bother people about fracking.”

(Green lender)

Another participant felt that natural gas was not “green” enough stating that biodiesel was a better, albeit underutilized, alternative:

“…as we increase our biodiesel level we will in fact be cleaner than natural gas at all levels.”

(City subcontractor supporting the Clean Heat Program)

Whether #2 is indeed cleaner than natural gas is challenging to assess. To our knowledge, no scientific literature has established differences in pollutants, i.e., PM, NO_x_, and CO_2_, specific to boiler transitions. Instead the literature has seemed to focus on mobile sources [29]. Furthermore, the ‘cleanliness’ of an energy source is typically judged by pollutants at the demand side of the energy spectrum. As alluded to by the green mortgage lender, some might suggest that a more holistic approach be taken to consider both supply and demand dynamics of environmental pollution, health, and justice consequences [30]. 

*Cost Considerations:* The process of conversion is complicated by the fact that boilers can have a 30+ years lifespan in relatively old buildings that are ill-suited for today’s heating technology. As a result, property owners and managers would have little familiarity with the financial or logistical considerations involved with heating oil conversions. Some landlords believed the process to be relatively straightforward,

“Going from 6 to 4 is relatively inexpensive, I can say around $5000. Going from 6 to 2, more expensive, could be as much as 15 to 20 thousand depending on the equipment.”

(Building owner)

A lingering contention between #2 and natural gas proponents is the price point.

“…last year at this time you were paying maybe $3.50 a gallon of oil, converting to gas you’re paying $2, you know, $1.70, $1.80.”

(Heating oil supplier)

The prices have indeed fluctuated over the years and, for different reasons, many stakeholders fear continued priced volatility in the natural gas market. 

*Going beyond the Clean Heat Call:* Energy efficiency is a worthy goal and the Clean Heat Program specified measures to incentivize its implementation. Numerous lenders were recruited into the Program in order to offer financing alternatives, and they explained having promoted energy efficiency whenever it was a financially viable strategy for building owners. In fact, one employee for a lender mentioned:

“When we do a loan we try to put energy efficiency or retrofits into every loan. We’re not a government program so we can’t force it, but we benchmark the building before we loan them the money…so we approach owners with common sense money saving approaches.”

(Green lender)

Another lender noted that prospective borrowers approached the banks for refinancing, and it was indeed the lender who mentioned the need to transition away from #6. The lender reported that this was a successful approach when applicable.

Collaborations were also vital to the success of many projects. Government and non-profit programs allowed the buildings to conduct efficiency upgrades in a cost-effective manner. The mainstream lender commented that:

“…the majority of our buildings were in conjunction with weatherizing the building with [weatherization organizations]… replacing windows, boilers, [etc.] can get very expensive, so having the help of [nonprofits] enabled us to do all of the work we wanted to do…”

[Traditional lender]

A community-based weatherization organization made sure to point out the importance of incentives and considering the property managers’ profits:

“There are very few building owners of multifamily buildings that are environmentalists. They are capitalists, they want to make money.”

(Building owner)

The process of converting home heating oils was, therefore, not necessarily intuitive to property managers and landlords. Indeed, costs and logistics of conversions were context-specific and require assessment by professionals who privilege the owner perspective and economic returns rather than the resident experience or justice concerns.

#### 4.2.3. Problems

*Lack of Information*: Building owners/managers that were required to convert to cleaner fuels varied in their understanding and justification for doing so. A consistent message expressed by almost all of the stakeholders suggested that landlords were unclear about their options and how to go about the conversion process. Respondent notes:

“…one of the biggest challenges is that buildings [owners] just didn’t quite understand, they understood that they needed to convert but they didn’t understand what their options were.”

(City subcontractor supporting the Clean Heat Program)

“…one of the things that we’ve also discovered is that nobody knows how a boiler works, and nobody knows what it actually takes to convert your boiler, if it even takes anything.”

(Representative from elected official’s office)

Landlords were often reliant on service professionals to provide pertinent information about the details of the policy and how to undertake the heating oil conversion.

“So the good news is I think at this point, hopefully nobody doesn’t know about the local law 43, because their boiler maintenance group, that service the boilers and the oil services groups who deliver the fuel have certainly, I think, been keeping people abreast of what they need to do… They generally will talk to a contractor about what their options are, [such as] is there gas availability?”

(Community-based non-profit outreach staffer)

These professionals provide technical services with regard to boiler upkeep and resupplying fuel, and while service professionals were an important source of information, the process remained confusing and risky for landlords. An outreach worker recounted a conversation with a landlord indicating:

“And one [property management] company, the guy told me that he gets confused by all of the different companies that are contacting him with services that are supposedly gonna help them meet or comply with these local laws, and at the same time save them money on their energy bills. So that was one of the things, he’s like ‘I got all these people sending me messages via email, via regular mail, and I don’t know which ones are actually trying to scam me, which ones are the real deal. I wish they could provide us a list of which companies are actually worth my time and energy as opposed to having to figure it out by trial and error.’”

(Community-based non-profit outreach staffer)

Similarly, stakeholders noted that some building owners were confused about how the three laws—one state and two local—worked, and how these regulations affected them. Landlords cited lack of knowledge, financial hardship, and bureaucratic red tape as critical barriers, a perspective also shared by those charged with supporting the transition. One respondent noted:

“…a lot of buildings that didn’t have the resources, or didn’t somehow get the message that they had to do something, or have realized that #4 is a short term solution that is not ideal.”

(City subcontractor supporting the Clean Heat Program)

While the policy was accompanied by numerous resources to help landlords, including financing and outreach support, landlords were often distrustful and found it difficult to identify credible sources. Property managers were often amenable to change, but were hampered by bureaucracy and resident politics to transition from heating fuel. Still, despite the fact that the Clean Heat Program was designed with financing tools and incentives to support building transitions to cleaner fuel sources, some property owners found that the options remained “prohibitively expensive.” 

*Inadequate Outreach:* The NYC Clean Heat Program contracted with non-governmental organizations (NGOs) for outreach and support efforts to help overcome various hurdles associated with the clean heat conversation. Incentivizing transitions was sometimes challenging when residents did not have the knowledge base or perspective to adequately weigh the options as noted by.one key informant who described working with a co-op board:

“…property managers really appreciate it when we go to co-op and condo boards since they have to vote on everything and it can be really difficult to get them to move. … they said they will not do it [transition heating fuel], it costs too much money… It’s difficult to get people to realize that it costs less to use grade 2 oil than to maintain a boiler using grade 6.”

(City subcontractor supporting the Clean Heat Program)

Multi-pronged outreach efforts were built into the Clean Heat Program to avoid misunderstanding and misinformation. Related resources were delivered by community-based nonprofits, consulting firms, banks, and contractors. As previously mentioned, some banks informed potential borrowers about the Clean Heat Program and described related financing incentives. However, multiple community-based nonprofits identified important challenges for outreach, noting that they did not provide goods or services, nor did they enforce any laws. This inability to compel compliance served as a challenge to some. Successful strategies to overcome this challenge included arrangement of building owners forums, which included a spectrum of participants. One interviewee who organized these forums with HPD recounted:

“…there were about 10 panelists from various non-profits and government agencies that do provide services for buildings. And that’s when a lot of property management companies and also coop shareholders came out…”

(Community-based non-profit outreach staffer)

Despite the success of these forums, a persistent and disappointing trend identified by outreach organizations was that many more buildings did not convert on time. One interviewee of a city-contracted consulting agency noted:

“…we’ve been tracking the data and seeing that buildings were converting and we saw that particularly rent stabilized buildings were converting more slowly.”

(City subcontractor supporting the Clean Heat Program)

These comments demonstrate that outreach may have been focused on the need for fuel transition, but less so on the mechanics/logistics of achieving conversion. Overall, interviewees agreed:

“Clean Heat resources that the city put together has been fantastic. I mean they’ve done a really good job just getting out there with outreach and education and targeting building owners…”

(Representative from elected official’s office)

Overall, the stakeholders identified numerous contextual factors that facilitated and detracted from the success of the program. However, qualitative analysis revealed that static elements were not the only issues of interest. Table 4 summarizes these findings. We found that processes of program implementation were integral to understanding the outcomes associated with the Clean Heat Program. Further, several problems hindered full and equal program implementation, including unsuccessful outreach efforts, and high costs associated with cleaner fuels. Despite these challenges, the program was an innovative and rather successful public-private partnership which achieved a near universal compliance with the #6 ban.

## 5. Discussion

The nearly universal compliance with the #6 ban is impressive given the scale and timeframe [31]. Despite several challenges, the program was an innovative and rather successful public-private partnership. While almost all buildings in NYC were in compliance with the ban of #6 fuel, many continue to burn residual fuel oil #4. These buildings are not in violation of the law, as #4 is set to phase out by 2030; however, #4 is disproportionality represented in buildings Uptown and it appears that incentives to switch them towards cleaner heating alternatives were unsuccessful. The analysis presented above, summarized in Table 4, demonstrates the Clean Heat Program has some key weaknesses that may have led to uneven adoption of clean fuels in Uptown, including unsuccessful outreach efforts, cost-prohibitive conversion opportunities, and (the perception of) a volatile energy market for clean fuels.

The stakeholder interviews elucidated the limited effectiveness of outreach efforts. However, it is possible that the financing initiatives were also not enough to catalyze conversions among those buildings needing the most resources, i.e., low- and moderate-income buildings. Although most buildings achieved compliance, it would seem that policies were only effective at motivating conversion away from #6, and not yet from #4, a particular environmental justice concern. The findings indicate that, despite outreach and education efforts, stakeholders may have lacked the appropriate incentives, knowledge, or resources to make this transition.

Overall, there was uncertainty among participants about the fuel market. Some stakeholders indicated that building owners fear the price tag of cleaner fuels, while others believed that the fuel markets may become more volatile in the future. This was complicated by contradictory knowledge/concerns regarding how clean natural gas is compared to diesel fuels and also considering the environmental impact of energy production. Furthermore, the variability in access to natural gas infrastructure affected transitions accordingly. 

### 5.1. Policy Implications and Future Directions

Air pollution is a nested policy issue, with levels of complexity and potential response. Historically, air quality advocates have focused on creating policies to curb ambient air pollution at the source by changing allowable fuel types, switching to more efficient technologies, etc. Consequently, these policies emphasize a source reduction approach. These source reduction strategies can be complemented with policies targeted at the building level. As policymakers consider potential energy futures, they should also consider the various legal, financial, and social incentive structures which facilitate and hinder source reduction and energy transitions. One such consideration at the building-level could be building energy rating systems. Implemented throughout much of Europe, these rating systems are publicly available, and thereby offer visibility to an often obscure problem [30]. Precedent exists for rating systems in NYC, wherein the NYC Department of Health and Mental Hygiene has used a publically-accessible grading system for eating establishments since 2010. This grading system has proven successful at improving sanitary conditions at restaurants [32]. In the case of the Clean Heat Program, transparency by way of a building energy rating system could empower building residents with information to hold landlords accountable to carry out clean energy transitions and incorporate energy efficiency, either through complaint or lease signings. This measure can influence real estate transactions, provide additional information for NYC housing consumers, and create a new surveillance mechanism for building operators. 

Although transition results appear mostly encouraging, any policy is not without its pitfalls or unanswered questions: Will natural gas prices stay low as the industry and market mature? Can the several thousand remaining #4 heating oil users be incentivized to transition more quickly? If not, at the expense of who’s health? How are the environmental impacts of production weighed against, or with, end-use? Furthermore, although observations and estimates indicate reductions in ambient pollutants, it is unclear the degree to which those reductions translate into improved indoor air quality. This is an important area of inquiry for future investigation. 

By many metrics, public health is improving for populations worldwide. However, those benefits are not always equal and disparities in health remain and, in some cases, are worsening. Our case study demonstrates that air quality is improving for all New Yorkers, but perhaps is not improving as much for those living in disadvantaged neighborhoods. While this case study focuses on NYC, our approach and premise is widely applicable. Indeed, little work has focused on how environmental inequities can persist through policy mechanisms. Global environmental health researchers can, and should, examine the upstream determinants of differential environmental exposures in order to design and implement more effective and equitable policies. 

### 5.2. Strengths and Limitations

In this study we sought to understand some of the dynamics that helped or hindered transition to clean-burning heating fuel oils at the time of the Clean Heat policy implementation. We believe that our mixed-methods approach is an asset, as it afforded us the opportunity to examine the demographic and geographic patterns of transitions while then interrogating some of the challenges of boiler change in Uptown. The key informants helped situate this transition in terms of the implementation process, however, the sample was small and did not include Uptown resident perspectives; the latter is the focus of future analysis. While our results may not be generalizable, the emergent, salient themes are valuable to environmental policymakers when considering large-scale energy transitions. Future efforts should continue to apply mixed methods approaches to comprehensively examine differential use patterns and equitable policy adoption of large-scale environmental policies. 

## 6. Conclusions

NYC continues to make great strides towards green and sustainable energy transitions, with Mayor DeBlasio acting to reduce carbon emissions 80% by 2050 [33]. In this paper, we have described the Clean Heat Program, its implementation, stakeholder perceptions, key successes and challenges, significant lessons learned, and future areas of inquiry. The main success is the near complete compliance with the ban of #6 fuel oil for boilers. With almost one million buildings throughout NYC, this is no small feat. Importantly, we uncovered critical gaps in the equitable transitions to clean heating fuel sources which has significant implications for environmental justice. Our novel analysis was meant to consider the ways in which implementation can be studied within the context of an environmental health policy, and for its equitable and just application. The analysis of the NYC Clean Heat Program serves as a case study of environmental health governance strategies and challenges. It is evident that broad scale environmental policies have the potential to improve public health when they are strategically designed and optimally implemented, with equity and justice as guiding principles. Therefore, we recommend that researchers interested in environmental justice consider studies that characterize how policies can yield different results across different communities. Understanding these mechanisms allows policymakers and environmental justice advocates to design, create, and employ policies and incentive structures that move society towards environmental and health equity.

## Figures and Tables

**Table 1:** New York City Air Quality Scores (2007 and 2017).

	2007			2017		
County (Borough)	Ozone Grade (A–F)	24-h Particle Grade (A–F)	Annual Particle Grade (Pass/Fail)	Ozone Grade (A–F)	24-h Particle Grade (A–F)	Annual Particle Grade (Pass/Fail)
Bronx	C	F	F	F	A	P
Kings (Brooklyn)	Data Not Available	F	P	Data Not Available	A	P
New York (Manhattan)	Data Not Available	F	F	D	A	P
Queens	D	F	P	F	A	P
Richmond (Staten Island)	F	F	P	F	A	Data Not Available

Adapted from American Lung Association’s State of the Air 2007 and 2017 Reports [2,3].

**Table 2:** Policies that comprise the NYC Clean Heat Program.

Name	Year	Government Level	Policy Type	Intent
New York State S.1145-C	2010	State	Law	Reduction #2 diesel oil sulfur limits: 2000 to 15 ppm
NYC Local Law 43-2010	2010	City	Law	Requirement of ≥2% biodiesel in home heating oils and reduction of #4 diesel oil sulfur limits: 3000 to 1500 ppm
Amendments to Title 15, Chapter 12 of the Rules of the City of New York	2011	City	Regulation	Ban of #6 diesel oil by 2015 and #4 by 2030

**Table 3:** Distribution of residual fuel burning buildings (#4 and #6) from 2011–2015.

Year	Uptown	Rest of NYC	Total
2011	47.9%	52.1%	7170
	(N = 3434)	(N = 3736)	
2015	53.2%	46.8%	3242
	(N = 1724)	(N = 1518)	

**Table 4:** Main qualitative findings.

Process	Problems	Successes
Compliance triggered by boiler permit renewals	Unsuccessful outreach efforts to building owners	Public-private partnerships were effectively executed
Public surveillance via “spot the soot”	High cost and delays with transition to cleanest fuels	Near universal compliance with the #6 ban
Financial support for building owners often coupled with weatherization efforts	Perception of a volatile energy market for clean fuels	
